# Identifying Opportunities for Early Detection of Cerebral Palsy

**DOI:** 10.3390/children11050515

**Published:** 2024-04-25

**Authors:** Brittany Hornby, Ginny S. Paleg, Sîan A. Williams, Álvaro Hidalgo-Robles, Roslyn W. Livingstone, Parma E. Montufar Wright, Alice Taylor, Michael Wade Shrader

**Affiliations:** 1 Physical Therapy Department, Kennedy Krieger Institute, Baltimore, MD 21205, USA; hornby@kennedykrieger.org; 2 Department of Physical Medicine and Rehabilitation, Johns Hopkins University School of Medicine, Baltimore, MD 21205, USA; 3 Independent Researcher, Silver Spring, MD 20901, USA; ginny@paleg.com; 4 School of Allied Health, Curtin University, Perth, WA 6009, Australia; sian.williams@curtin.edu.au; 5 Liggins Institute, University of Auckland, Auckland 1023, New Zealand; 6 Facultad de Educación, Universidad Internacional de La Rioja, 26006 Logroño, La Rioja, Spain; alvaro.hidalgo@unir.net; 7 Occupational Science and Occupational Therapy, University of British Columbia, Vancouver, BC V6T 2B5, Canada; roslyn.livingstone@ubc.ca; 8 Nemours Children’s Health, Wilmington, DE 19803, USA; pwright@udel.edu (P.E.M.W.); alice.taylor@nemours.org (A.T.)

**Keywords:** early diagnosis, early intervention, evaluation, assessment, MRI, GMA, HINE, neurodevelopment

## Abstract

This study aimed to evaluate assessment and referral practices for the early detection and diagnosis of children at risk for or with cerebral palsy (CP) by health care and education providers in Maryland and Delaware. A secondary aim was to identify barriers for using early detection tools and identify opportunities for change to support early diagnosis and improve care. Seventy-two participants answered ≥ 50% of the survey questions. Most were occupational or physical therapists (86%) working in early intervention (61%). Eighty-eight percent indicated awareness that CP can be diagnosed by 12 months. Though 86% stated they typically suspect a diagnosis of CP between 0 and 12 months, only 19% reported that their patients received a CP diagnosis < 12 months. The Developmental Assessment of Young Children (73%) and the Peabody Developmental Motor Scales-2 (59%) were used most. Many respondents indicated never using magnetic resonance imaging (70%), the General Movements Assessment (87%), or the Hammersmith Infant Neurological Exam (69%). Participants identified clinical signs and symptoms prompting a referral for the diagnostic assessment of CP, most commonly stiffness in legs (95%), excessive head lag (93%), and persistent fisting (92%). Policy and organizational change, clinician education, and training are needed to support the implementation of CP early detection guidelines.

## 1. Introduction

In recent years, a growing body of literature, including systematic reviews [1,2], has underscored the pivotal role of clinical tools in the early detection of infants with or at risk for cerebral palsy (CP). Notable among these tools are magnetic resonance imaging (MRI), Prechtl General Movements Assessment (GMA) [3], and the Hammersmith Infant Neurological Examination (HINE) [4,5]. Evidence strongly supports the implementation and timely use of these key diagnostic tools, indicating that the age of CP diagnosis can be below 12 months and closer to 5–6 months [2,6].

Furthermore, the implementation of international guidelines for early detection has been shown to decrease the age at CP diagnosis by 7.5 months (average diagnosis age of 11.6 months) [7]. It is also possible to predict which children with CP are at highest risk of being non-ambulant (Gross Motor Function Classification System (GMFCS) level IV or V) using GMA Motor Optimality Score (2–5 months) [3] and HINE (3–24 months) [4] cut-off scores.

In addition to providing families with early answers, clarity, and support for their child’s condition [8], early diagnosis provides the opportunity for early intervention (EI) and management to optimize a peak period of “infant motor and cognitive plasticity and to prevent secondary complications and enhance caregiver well-being” [2]. The first step of the implementation efforts involves a planning/preparation phase, assessing the use of cited tools as well as enablers and barriers to the early detection programs [9]. To this end, surveys have been completed in the United States (US) [10], Spain [11], and New Zealand [12], with this information being used by local providers to help advocate for increased education, training, and improved resources in clinical practice.

The exploration of the regional adoption of early detection practices has revealed potential asymmetrical implementation, influenced by local policies, health care systems, or referral pathways, among other regional specifics. This variability in adoption rates and practices underscores the need for a comprehensive understanding of regional disparities, as exemplified by the varying frequencies of tool usage across different Spanish communities (with higher frequencies in the communities of Madrid, Basque Country, and Asturias) [11].

The only published US survey to date [10] focused on intervention strategies that occupational therapists (OT) and physical therapists (PT) use with infants with or at risk for CP. It identified that only 4% of providers always used the GMA for early detection of CP, and 0.7% used the HINE to determine motor severity. No analysis was conducted regarding any regional differences. There are no studies demonstrating how the recommendations for the early detection of infants with and at risk for CP are being implemented in the states of Maryland or Delaware. These are small adjoining states in the lowest part of the northeast US and bordered to the east by the Atlantic Ocean. Establishing a baseline of current practice and contact (i.e., location/site and service) and specific opportunities for change in practice is imperative to guide future planning for the successful implementation of best practice recommendations [13].

Therefore, this study aimed to evaluate current practices regarding assessment and referral for early detection and diagnosis, and referral practices of health care and education providers in the states of Delaware and Maryland for young children, birth through 3 years, who are at risk for or diagnosed with CP. A secondary objective was to identify barriers to the use of key early detection tools (such as MRI, GMA, HINE, and clinical signs/symptoms), including barriers for use, to identify possible opportunities for change in practice to improve early detection and early management.

## 2. Materials and Methods

### 2.1. Design and Recruitment

This descriptive, cross-sectional survey study included a convenience sample of health or education professionals providing services to children from birth to 3 years of age, with risk factors for CP, within the states of Maryland and/or Delaware, US. Ethics approval was provided by the Nemours (Wilmington, DE, USA) research ethics board (IRBnet #: 2043288). Recruitment specifically targeted physiatrists, pediatricians, neurologists, orthopedic surgeons, nurses, PTs, OTs, and other EI health or education professionals. Individuals working in states other than Maryland or Delaware, those who do not provide services for children under age 3 years, and those who do not read or understand English did not meet inclusion criteria.

Participants were recruited via email either directly or through snowball sampling by the following methods: EI provider email contact lists for Maryland and Delaware, directors of EI services within every county in each state were asked to distribute to relevant staff members, researchers forwarded the survey link to EI or medical contacts within their institutions or professional circles, relevant professional associations were contacted and asked if they were willing to forward to their membership within Maryland and Delaware, and individual respondents were asked to forward the link to their eligible colleagues.

### 2.2. Survey Instrument

Similar survey studies regarding EI professionals’ attitudes, knowledge, and use of tools and methods for the early detection of CP have been previously conducted in New Zealand [12] and Spain [11]. The survey used in this study was adapted from these previous surveys with permission and assistance from the authors. Items were generated based on study objectives, literature review, and results of previous survey studies. They were then refined through discussion by investigators, with input from external clinicians currently involved in diagnosis and/or intervention with young children at risk for or diagnosed with CP.

The questionnaire was semi-structured, with both closed and open-ended questions. A maximum of 25 questions were included to minimize respondent burden. “Display logic” was used to customize questions based on respondents’ answers. For example, individuals who indicated they never used a particular tool would be asked about reasons for non-use, while those who did use the tool would be asked further questions about scoring. The survey was piloted by a group of three expert clinicians. Researchers then refined survey questions and response formats to improve clarity, reduce redundancy, and ensure that included questions were priorities and addressed research objectives.

The final survey covered three general areas (see Supplementary File S1 for the survey tool):

1. Demographics: professional background, location, experience, workplace setting, and EI caseload.
2. Early detection/diagnosis: current practice for CP diagnosis and/or referral for CP diagnosis and use and knowledge of specific screening tools and methods.
3. Future directions for knowledge translation: barriers and facilitators to use and implementation of tools and methods for early detection of CP.

### 2.3. Data Collection

Potential participants were provided an email link to complete a voluntary online survey via Research Electronic Data Capture (REDCap) [14]. The survey took approximately 15 min to complete. Eligibility criteria were provided at the start of the survey, and participants were informed that no identifiers would be collected, and their responses were anonymous. By continuing to the survey questions, participants provided informed consent to be part of the research study.

Those receiving the email link were invited to participate and/or forward the link to other eligible colleagues. The survey link was initially disseminated by email on 18 July 2023, to 107 contacts (73 in Maryland, 34 in Delaware). Follow-up reminders (after 1 week, 3 weeks, and 6 weeks) were sent out following the initial email to maximize participation. In addition, monthly email reminders were sent to county EI programs in Maryland. After 3 months, EI leaders from counties with zero responses were contacted by telephone and asked to forward the link to their staff. An additional 20 links were sent in November 2023 directly to individual EI therapists in counties lacking responses. The survey closed on 1 December 2023.

### 2.4. Data Analysis

Data were exported from REDCap into Microsoft Excel for Microsoft 365 MSO (version 2312 Build 16.0.17126.20190) for analysis. Frequencies and percentages were calculated to provide a descriptive analysis of the data and organized according to study objectives. Graphs and charts illustrating visual analysis were created using Microsoft Excel and Canva (www.canva.com, accessed on 15 January 2024).

Descriptive content analysis of free-text answers was conducted by three authors (GSP, AHR, RWL). A deductive/directed analysis approach was taken [15] using the categories identified by Williams and colleagues [12] and adapted by Merino and colleagues [11] to allow for the comparison of findings with these previous studies. All free-text answers were coded under the themes system factors, social factors, health provider knowledge and perceptions, and clinical considerations. Themes and sub-themes are detailed in Supplementary Tables S1 and S2, and highly reported themes are discussed in the manuscript.

## 3. Results

**Figure 1:**
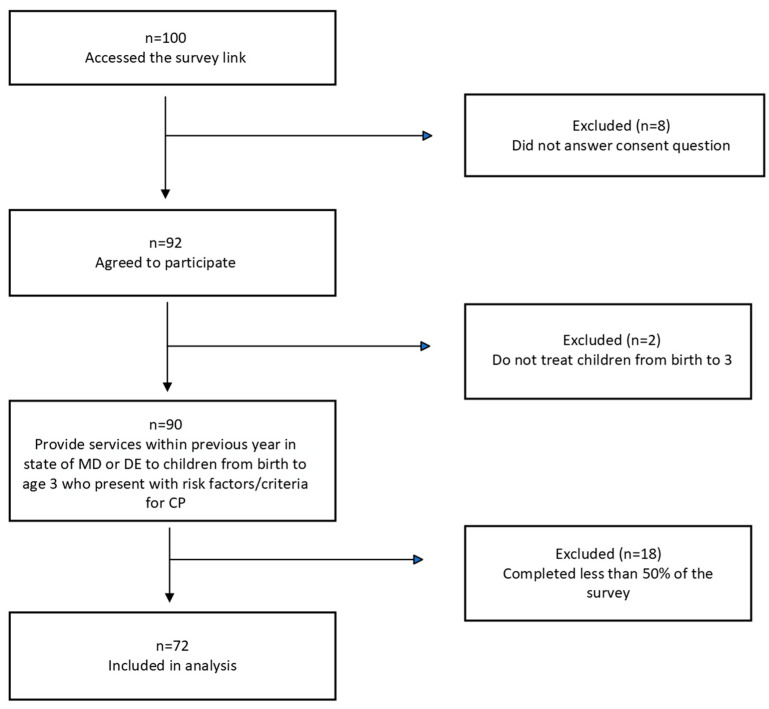
Summary of included survey responses.

A total of 100 individuals accessed the survey link. Figure 1 illustrates the inclusion and exclusion of responses, with a total of 72 survey responses being included in the analysis.

### 3.1. Participants

**Figure 2:**
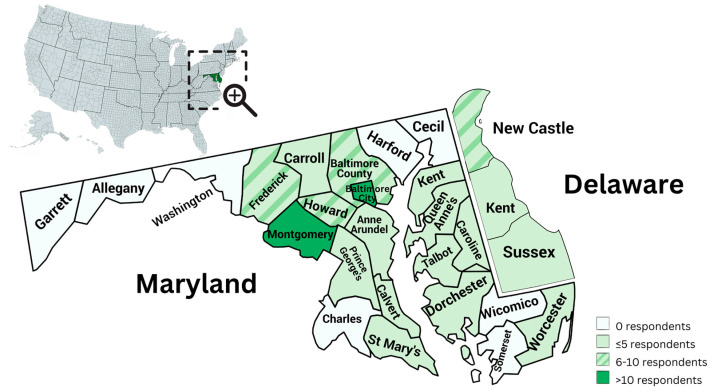
Distribution of respondents within Maryland and Delaware counties.

A total of 72 health and education professionals answered at least 50% of the survey questions. Seventy-nine counties were identified as current places of employment, with 68/79 (86%) being employed in Maryland and 8/79 (10%) in Delaware. Of note, in the survey, a single option for Kent County was listed, so it is unknown whether the two participants who provide services in Kent County do so within Maryland or Delaware. One out of 79 respondents indicated that they worked in a county other than those listed. Figure 2 illustrates the position of Maryland and Delaware within the US, and the distribution of respondents within each county.

Seventy-two individuals reported their professions, with two reporting multiple professions. Most respondents were PTs (*n* = 36, 48.6%) or OTs (*n* = 18, 24.3%). Of the 71 respondents who indicated length of time practicing their profession, over 70% had more than 15 years’ experience. Respondents worked in multiple settings, with 108 settings reported by 71 individuals and between 8% and 100% of their caseload consisting of children from birth to 3 years old. Most participants (43/70; 61.4%) reported seeing between 1 and 5 children with CP under 3 years of age 3 in a typical month.

A total of 66 respondents provided 76 responses in relation to professional memberships, although only half (33/66) were members of any professional society. National OT and PT association memberships were most prevalent, while only six were members of the American Academy for Cerebral Palsy and Developmental Medicine, and three were members of the Child Neurology Society. Other society memberships reported included National Education Association, International Association of Infant Massage, Pediatric PT Residency Group, American Neurological Association, American Academy of Neurology, Newborn Brain Society, Council for Exceptional Children, and American Academy of Pediatrics. See Table 1 for a summary of respondent characteristics.

### 3.2. Age of CP Diagnosis

Eighty-eight percent (*n* = 57/65) of respondents indicated that they were aware that CP can be diagnosed by 12 months of age. However, only 18.8% (*n* = 13/69) reported that children in their care receive a CP diagnosis by this age compared with 50.7% (*n* = 35/69) at 13–24 months and 27.5% (*n* = 19/69) at 25 months to > 36 months. Eighty-six percent of participants (*n* = 61/71) indicated that they typically suspect CP within the first year without sharing this with the family or formally giving a diagnosis. Of these 61 respondents, only 11.5% (7/61) can provide a diagnosis within their scope of practice, and most (39/61; 64.0%) work in EI/infant and toddler programs.

Many free-text answers (*n* = 15) in relation to age of diagnosis suggest that health professionals consider diagnosis to be delayed or late in Maryland and Delaware.

*The doctors tend to be reluctant to diagnose CP early and will usually say “Let’s wait and see”.*

—ID7

The timing of diagnosis is influenced by severity, with diagnosis reported as being most delayed for children with milder presentation.

*Depends upon the severity. There are kids who suspect at 0–6 mos given their presentation and history and may receive a diagnosis that early or 7–12 mos, but more often it is not until kids are not walking by age expected milestone.*

—ID 31

System factors such as referral and health pathways influence timing of diagnosis and were frequently mentioned (*n* = 20).

*Delays in or barriers to getting genetic testing or getting sedated MRI may contribute to perceived delayed diagnosis.*

—ID53

Social factors such as family readiness were raised, but respondents reported opposing viewpoints.

*The longer diagnosis is deferred, the more difficult for parents and family.*

—ID49

*Working on improving—balancing goal of early diagnosis with individual family need /readiness (without limiting therapies/support).*

—ID34

Health provider knowledge and perceptions were raised by 12 respondents. One respondent was frustrated with doctors suggesting more hands-on therapy to “fix” the problem rather than diagnosing and recommending CP-specific resources.

*I wish MDs would identify earlier and trust providers … stop recommending more of the same therapy and identify resources.*

—ID41

A contrast between the views of two “diagnosing” professionals was also revealed, with one recommending early diagnosis and the other revealing a bias against the use of the CP label or recognition of the spectrum of functioning.

*I like to diagnose or at least educate families on the possible diagnosis of CP so they can get the correct services in place.*

—ID37

*“CP” is such a broad swatch. For the mildest I tend to use another label in medical*

*communications because of the potential for misinterpretation of its impact on functioning.*

—ID48

Additional quotations and details may be found in online Supplementary Table S1.

### 3.3. Awareness and Use of Assessment Tools

**Figure 3:**
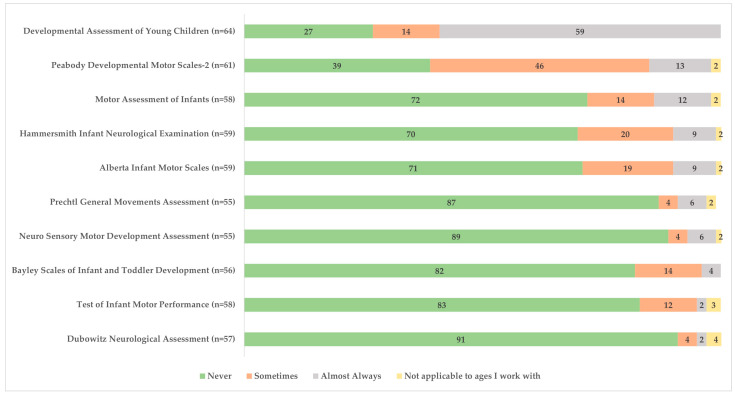
Frequency of assessment tool use for children with or at risk for cerebral palsy among health professionals (expressed as % respondents).

Figure 3 illustrates tools used for the birth to 3 years old population who are at risk for CP. The most used assessment tools were reported to be the Developmental Assessment of Young Children and the Peabody Developmental Motor Scales-2. In reference to the highlighted/recommended use of MRI, GMA, and the HINE for early detection [2], 70.2% (*n* = 40/57) indicated they had never used (or referred to) MRI, 87.3% (*n* = 48/55) had never used the GMA, and 69.5% (*n* = 41/59) had never used the HINE.

When asked why a provider did not use or refer a child under the age of 1 for an MRI (*n* = 37), the most common responses included other reasons (*n* = 22), my workplace does not support it (*n* = 11), and it is not familiar to me (*n* = 3). The most reported reasons for not using the GMA included not knowing how to administer/score it (*n* = 32/48), the GMA not being supported by their workplace (*n* = 12/48), and a reliance on clinical signs/symptoms (*n* = 10/48). The most reported reasons for not using the HINE were not knowing how to administer/score it (*n* = 29/41), a reliance on clinical signs/symptoms (*n* = 12/41), and the HINE not being supported by their workplace (*n* = 10/41). Details on the non-use of the GMA or HINE are outlined in Table 2.

Of the five providers who indicated that they did use the GMA, one respondent indicated that they knew how to assess general movements within the writhing period, and three respondents indicated they knew how to identify abnormal/absent fidgety movements at 2–5 months adjusted age.

Of the 17 providers who indicated that they did use the HINE in practice, further questions were asked about what they do next with the HINE score: “I know how to look up the score and see if a kid is at risk for CP or not” (*n* = 10), and “I know how to get the total score” (*n* = 5). Only three selected “I know how to predict GMFCS at age 2 years”, and seven respondents reported that they were familiar with the tool but not the scoring. Of the 17 providers who reported using the HINE and/or GMA in practice, most (14/17) were PTs, worked in EI/infants and toddlers (9/17), report that children in their care typically receive a diagnosis of CP between age 13 and 24 months (11/17), and most commonly refer to developmental pediatrics if they suspect that a patient may have or be at risk for CP (10/17). The most common signs and symptoms that these providers use to prompt referral for diagnostic assessment of CP are stiffness/tightness in legs between 6 and 12 months (13/17), any asymmetry in posture or movement (11/17), abnormal reflexes (10/17), and persistent fisting in babies older than 4 months (10/17).

**Figure 4:**
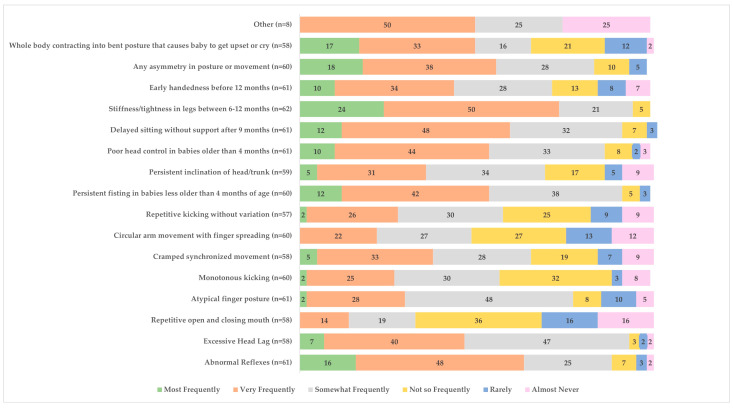
Clinical signs used to help detect children at risk for or diagnosed with cerebral palsy (%).

Common clinical signs and symptoms that were reported by all survey respondents to be used (i.e., using frequently, very frequently, most frequently) to assist with detecting children at risk for or diagnosed with CP included abnormal reflexes (54/61), excessive head lag (54/58), atypical finger posture (47/61), persistent fisting in babies older than 4 months (55/60), poor head control in babies older than 4 months (53/61), delayed sitting without support after 9 months (55/61), stiffness/tightness in legs between 6 and 12 months (59/62), and any asymmetry in posture or movement (51/60). See Figure 4 for details.

Eight respondents indicated that they use other signs and symptoms that were not part of the survey including seizures, nystagmus, hypertonicity, any other upper motor neuron finding in constellation with history/birth and prenatal factors and developmental assessment, abnormal motor development, and atypical torticollis.

### 3.4. Referral Pathways

When asked “if you suspect that a patient may have or be at risk for CP, what is your usual referral pathway”, 71 participants provided a total of 139 responses. The most common referral pathway was to pediatric neurology (*n* = 37/71, 52.1%), developmental pediatrics (*n* = 34/71, 47.9%), general pediatrics (*n* = 20/71, 28.2%), and physical medicine and rehabilitation (*n* = 18). Other providers referred to included therapists (PTs, OTs, speech-language pathologists; *n* = 17), pediatric orthopedics (*n* = 6), and others (*n* = 4).

The three respondents who did not typically make referrals for diagnosis either worked in a clinic where children were already connected with the appropriate services or where referring them on was the role of another professional (i.e., a PT or OT). Only a few respondents (10/70; 14.3%) reported that their workplace had guidelines or procedures for referrals related to early detection of CP, while many (34/70; 48.6%) specifically stated that no such guidelines were available. Others reported needing to refer back to the pediatrician, and one respondent suggested that a set guideline would not be relevant for all children’s needs. Following referral, 40/69 respondents (58.0%) indicated that the patients’ wait time for an appointment is more than 3 months.

### 3.5. Enablers and Barriers to the Implementation of New Tools or Procedures

#### 3.5.1. System Factors

Considerably more barriers (*n* = 44) than enablers (*n* = 13) were identified. Funding, time, workload and staffing, and availability of tools were reported as either barriers or enablers, while organizational policies appeared only as barriers.

Funding:

✓Enabler—*Support from admin to purchase and provide training, focus/practice sessions.*

—ID35

✗Barrier—*Cost of training for testing and testing materials cost and time to take training courses.*

—ID29

Time and workload and staffing:

✓Enabler—*Training and practice prior to implementing.*

—ID56

✗Barrier—*Time for training, resources for training, workload, leadership education regarding assessment tools.*

—ID30

Availability of tools:

✓Enabler—*We have access to many assessment tools and can choose which one we want to use.*

—ID97

✗Barrier—*Use of only two tools to determine eligibility, neither of which adequately address the movement patterns of babies under six months.*

—ID72

Organization policies: many barriers were described in relation this theme:

*Early intervention is through the school system. It is a large bureaucracy, and changes are made very slowly. Plus, the people making the decisions are not always the ones that have the most knowledge. We have changed our assessment tools multiple times.*

—ID83

*We are mandated by the state to use specific screening tools for eligibility so other assessments are typically done during PT visits or after the initial evaluation due to time constraints.*

—ID80

#### 3.5.2. Social Factors

In contrast, more enablers (*n* = 25) than barriers (*n* = 21) were identified under this theme.

Administration/leadership:

✓Enabler—*Team leaders for OT and PT or program director.*

—ID16

✗Barrier—*The state and other leaders within the organization that don’t understand what the PT’s role is in early intervention and requiring one particular assessment tool over another.*

—ID7

Peer/multidisciplinary/clinical champion interactions and support:

✓Enabler—*Engagement with other PTs and OTs through my employer and through my involvement with the MSDE OT/PT Steering Committee.*

—ID45

✗Barrier—*Would need the team to work together to change practice- would need an algorithm and help with scheduling.*

—ID37

#### 3.5.3. Health Provider Knowledge and Perception

Access to training, knowledge sharing and knowledge/confidence/practice, and guidelines/pathways were all reported with similar numbers of enablers (*n* = 22) and barriers (*n* = 19)

Access to training:

✓Enabler—*Trainings, access to appropriate tools, access to providers comfortable with tools and practices that I can use as a mentor for new knowledge/skills/tools.*

—ID36

✗Barrier—*Lack of access, training, or mentorship on these tools/topics.*

—ID36

Knowledge sharing/knowledge/confidence/practice opportunities:

✓Enabler—*Inservices, trainings from other departments such as NICU clinic and neurology.*

—ID94

✗Barrier—*Lack of training in some of the new assessment tools.*

—ID55

Guidelines and pathways:

✓Enabler—*Recommendations from AAP, CDC, and other similar organizations State Regulations.*

—ID88

✗Barrier—*The many steps one needs to go through to be OKed to use a specific assessment tool.*

—ID47

#### 3.5.4. Clinical Considerations

A small number of comments (*n* = 6 enablers and *n* = 4 barriers) pertained to clinical considerations in relation to tools or the internal drive and motivation of the clinician.

✓Enabler—*Ease of administration/scoring, application to diagnostics/treatment planning, real life functional information for parents, not cost prohibiting.*

—ID100

✗Barrier—*Trademarks or restricted use/pay for use is a barrier to getting it programmed to electronic charting. Getting scoring programmed or norms programmed so entering data, scoring it, then referencing it compared to norms could be seamless in Epic—those are barriers.*

—ID53

Additional quotes and all themes and sub-themes may be found in online Supplementary Table S2.

### 3.6. Other Topics Raised by Respondents

Additional free-text comments at the end of the survey were generally supportive of the project and advocated for early detection. Several comments were grouped under the theme health provider knowledge and perceptions.

One comment illustrates bias against the CP diagnosis:

*Because it is a spectrum disorder, I am worried that the label diagnosis is itself a barrier to quality care/caring.*

—ID48

While another suggests a lack of understanding that CP is a clinical description and may be accompanied by a genetic or other diagnosis:

*Many diagnoses present with similar motor presentations, including genetic disorders. It can be hard to separate this out or categorize under CP or something else.*

—ID35

In addition, one comment suggests that some perceive limited benefit from the use of new tools such as the HINE or GMA and are resistant to change:

*I feel that my clinical observations and neurological assessment are more valid than these standardized tests.*

—ID49

See Supplementary Materials for further result details.

## 4. Discussion

Survey results suggest that the diagnosis of children at risk for CP is frequently delayed in Maryland and Delaware. Only 19% of respondents report that children on their caseload receive a diagnosis within the first year, despite most (86%) suspecting CP before 12 months. Likewise, 60% of families have described being concerned about their child’s development before 6 months but still face delays in obtaining a diagnosis [16]. In this study, some professionals recommended early diagnosis, while others were concerned about family readiness. However, the need for early diagnosis for children at risk for or with CP has been strongly highlighted by families [8]. Early detection is crucial for starting early, specific, and evidence-based interventions [1,2], and plays a role in reducing stress for families [16].

A free-text answer option was added in this survey to allow more in-depth exploration of factors influencing age of diagnosis in Maryland and Delaware. In contrast to survey results from Spain, where most PTs reported CP diagnosis being provided around 12 months, only 18% of respondents reported diagnosis being provided for children on their caseload by this age. System barriers including lengthy delays for diagnostic appointments were highlighted in our survey, whereas both the New Zealand and Spanish surveys highlighted lack of clear guidelines and referral pathways for children “at risk” for CP.

This comparative analysis reveals a shared global challenge in early CP detection: the gap between the availability of sensitive and specific diagnostic tools [6] and their practical implementation in clinical settings. This issue has been well-documented in research, which often points out that it takes an average of 17 years for research evidence to be incorporated into clinical practice [17]. Given this significant delay, there is an urgent need for targeted strategies aimed at improving health research translation.

Guidelines for early detection of CP [1,2] have been available for a few years; implementation is reported to be feasible, and the potential of stepwise processes [9] suggests a feasible pathway for bridging this gap. In the US, successful strategies have been documented, ranging from the implementation of the HINE [18] to single-site experience of international guidelines [19] to a broader network implementation across five hospitals in California, Ohio, Utah, Texas, and Maryland [20].

However, survey results suggest that the recommended tools for early detection are not widely used in Maryland and Delaware. When compared with the previous survey studies conducted in New Zealand, the GMA and HINE were never used by 74% and 51% whereas in Spain, the GMA and HINE were never used by 62% and 60%, respectively. In this study, 87% of respondents reported never having used the GMA, and 66% of respondents reported having never used the HINE. Of only 17 respondents who did report using the GMA and/or HINE, 14 of these were PTs who may use it as a screening tool, but typically do not use it for diagnostic purposes in the US.

Although families in the US express a desire for factual discussions about the need for neuroimaging assessments as early as possible [8], this survey reveals limited use of MRI (70% never used or referred for use). These findings are not unexpected given the low percentage of individuals participating in this study who are able to diagnose (17.5%). However, this trend is not unique to our study; similar findings have been observed in other US studies, where 70% of health care providers (primarily physicians) rarely or never prioritized MRI immediately during their discussions with parents [8]. This observation warrants further study given the key role of neuroimaging in the early diagnosis triad: MRI, GMA, and HINE [6].

In contrast to the low percentage of potentially “diagnosing” professionals in our survey, 34% of participants in the New Zealand survey reported providing a diagnosis or detecting whether a child was high-risk for CP. The recommendation rate for MRI for “diagnosing” professionals varied from 81% in children under 1 year to 92% in those over 2 years, while 57% of “non-diagnosing” professionals never referred for MRI [12].

Lack of training was the most reported reason for non-use of recommended early detection tools in this survey and was also highlighted in the Spanish survey [11]. This issue was compounded by system barriers such as “time, workload, and staffing” (lack of time for updating/to conduct assessment), “funding” (for training and mentoring) and “organizational policies.” Similarly, “time, workload, staffing and funding” barriers were also identified in both previous surveys [11,12].

Despite more enablers being identified than barriers, significant challenges were also associated with social factors (including lack of “management/administration support”, “non/collaborative teamwork”, or poor “communication within/between services”) and clinicians’ knowledge of the assessment tool. Regarding the latter, it has been suggested that health providers’ negative perceptions or discomfort with the provision of early diagnosis may be related to their level of confidence in the reliance on a tool such as GMA [21] and concern for adding (potentially undue) stress on a family.

This barrier highlights a global need for improved and accessible educational programs and resources, continued professional development, and knowledge sharing. Standardized training and implementation of the HINE has been developed in both large high-risk infant follow-up [18] and local EI services [22]. The benefits of a short and tested training extend far beyond the practical use and inter-observer reliability of such tools. They have the potential to significantly elevate the awareness and knowledge of professionals regarding tools and referral protocols, directly influencing clinicians’ perspectives and actions [22]. Mentioning and explaining diagnostic tools aligns more closely with family needs, as identified in qualitative research (where 77% of providers rarely or never mentioned GMA or HINE) [8].

Regarding the GMA, its high diagnostic accuracy for infants at high risk has been well-documented in follow-up studies [23]. However, its accuracy in community settings, which reflects the reality for many survey respondents, presents lower values that need to be studied [24]. Moreover, it has been shown that inter-observer reliability increases with the observer’s experience with the tool [25]. This suggests that perhaps training alone might be insufficient; there is also a need for facilitated mentorship and peer review among clinicians. Such an approach has been highlighted in the surveys as beneficial, promoting interactions and support from peers and clinical champions or knowledge brokers.

The hesitation to adopt newer diagnostic methods could indicate a deeper organizational resistance to change, increased by policy barriers. These barriers are further complicated by state/region-level variations in the criteria for qualification for services. In the US, officially approved developmental assessment tools require age equivalent scores since, to qualify for services, children should be 25–50% delayed. Children may also qualify for services secondary to a diagnosis of CP or a genetic syndrome; however, the survey results suggest a significant delay in these diagnoses for many children. Children who presented with clear neonatal risks (e.g., born premature, low birth weight, or had intraventricular hemorrhage or hypoxic-ischemic encephalopathy, etc.) may also be qualified as “automatically eligible” or “atypical”.

These protocols, while resembling those in countries like Spain, only apply to a subset of children likely to be diagnosed with CP. More than half of children with CP are born full-term and have uncomplicated births [26] falling into the “typical” category, which may not immediately qualify them under high-risk follow-up, particularly if they function at GMFCS I or II. Furthermore, in states where children must be 50% delayed in two or more developmental domains, children are very unlikely to qualify for any services before 2 years of age. This is very concerning because current recommendations reinforce the importance of EI [1]. Free-text answers illustrated a lack of knowledge regarding clinicians’ abilities to use other tools, such as GMA or HINE, in addition to the approved tools used to qualify children for EI services. Time and workload barriers may also limit therapists’ use of tools in addition to the ones approved for service qualification.

Organizational barriers were frequently raised in this study, and reference was made to many EI services being funded under education or the local school district. The tension between education policies and organization systems and health care providers may increase difficulties in introducing new tools to detect CP. CP is not one of the diagnoses typically tracked in the education system and this may be an added barrier. It may be an explanation of potentially increased difficulties in early detection in the US, in comparison to Spain and New Zealand, where therapists working with young children at risk for CP are predominantly funded via the health care system.

As is the case in New Zealand and Spain, early detection of clinical signs and symptoms plays a key role in the screening of infants, whether they have newborn detectable risk factors or not. Clinical features identified in the literature as “prompt referral for diagnosis” [27] were also reported to be used frequently, very frequently, or most frequently. These features include hand preference before 12 months of age, stiffness or tightness in the legs between 6 and 12 months, hands fisted, head lag, consistent asymmetry of posture beyond 4 months, and delayed sitting beyond 9 months. Additionally, abnormal reflexes were also identified as “warning signs”; however, only the persistent startle (Moro) reflex has been specifically considered as such.

### 4.1. Recommendations for Clinical Practice and Future Research

A significant barrier to the widespread implementation of the GMA and HINE is the limited availability of formal training and certification programs. Although current leaders of HINE acknowledge the necessity for regular certification and fidelity retesting of physicians and diagnosticians, there appears to be an unmet need for a lower-tier cadre of “screeners”. These individuals would receive informal (potentially virtual) training on the HINE, enabling them to share findings with qualified medical professionals and expedite access to a specialty physician. Crucially, the currently employed training videos, deemed inadequate for formal training purposes, are inaccessible behind a paywall. We propose granting free access to these videos for the education of “screeners”, accompanied by a standardized proforma incorporating cut-off scores and GMFCS level approximations.

As part of the translation and cultural adaptation of the HINE to the Spanish context, the effective results of training have been demonstrated [13]. Eleven therapists (OT, PT, speech language pathologists and psychologists) who were provided with 4 h of HINE training adapted from the modules developed in the US [19] demonstrated excellent intra- and inter-rater reliability values, 1 year post training. Compared with pre-training scores, therapists increased their knowledge and confidence regarding the purpose, use, and interpretation of HINE scores. They also reported greater confidence in including parents in the assessment process, in communicating results to families and making appropriate referrals to specialists for diagnosis [13]. Increasing accessibility of the training videos to therapists in the US may have similarly beneficial results that could be demonstrated through the use of similar pre- and post-training surveys.

This survey provides some limited results regarding the current status of early detection and diagnosis of CP in Maryland and Delaware. Surveys would be needed in other states or on a national level to determine the status across the US. If the federal Department of Education added “cerebral palsy” to the current list of tracked diagnoses, this would greatly increase ability to measure each state and program’s progress. This would facilitate future research regarding implementation of early detection of CP and inclusion of children, particularly those at GMFCS I or II, in EI programming. A change such as this might also facilitate access to and use of the recommended early detection tools of GMA and HINE, in addition to the recommended tools used to determine percentage of developmental delay, addressing some of the policy, organizational, and management support barriers highlighted in the survey.

The survey used in this study is provided as a supplementary file to facilitate similar future studies in other regions. We would suggest the following changes based on our results: consider methods appropriate to the location that would increase physician engagement and methods to facilitate participation from more remote/rural counties.

### 4.2. Study Limitations

Although it is not possible to calculate a response rate due to the varied recruitment methods, the generalizability of these study results may be limited by the relatively low number of respondents and the uneven participation rates across the different counties. In addition, nearly all our respondents are typically not able to provide a diagnosis of CP within their professional role, and the responses from the few physicians who did participate may not adequately reflect the attitudes of those providing a CP diagnosis in Maryland and Delaware. Although it is possible that those choosing to participate in the survey may have been positively biased toward early detection of CP, skewing our results, quantitative and qualitative results suggest that respondents with differing attitudes and familiarity with early detection and tools were included.

## 5. Conclusions

This survey, conducted in Maryland and Delaware, illustrates limited use of recommended early detection tools and is consistent with results of previous surveys from New Zealand and Spain. Quantitative and qualitative responses suggest the need for federal, state, and local awareness of the importance of early detection and intervention for children at risk for or with CP. National, regional, and local policy changes are needed to support clinician training and ongoing peer mentoring.

## Figures and Tables

**Table 1:** Respondent characteristics.

Variable	Characteristics	Frequency	Percentage *
Profession (*n* = 74) (71 individual respondents)	Developmental pediatrician ^#^	1	1.35
	General pediatrician ^#^	3	4.05
	Orthopedic surgeon ^#^	1	1.35
	Pediatric neurologist ^#^	3	4.05
	Physiatrist ^#^	1	1.35
	Medically complex pediatrician ^#^	2	2.70
	Nurse practitioner/physician assistant ^#^	1	1.35
	Early childhood nurse	2	2.70
	Occupational therapist	18	24.32
	Physical therapist	36	48.65
	Researcher	1	1.35
	Early intervention administrator	1	1.35
	Social worker	1	1.35
	Special educator	2	2.70
	Speech-language pathologist	1	1.35
Years of experience (*n* = 71)	6–14 years	21	29.6
	15+ years	50	70.4
Workplace setting (*n* = 108)	Early intervention/infants and toddlers	44	40.74
	Schools (education services)	12	11.11
	Outpatient-based community hospital centers	11	10.19
	Outpatient-based academic hospital centers	9	8.33
	Private practice	7	6.48
	NICU follow-up program	7	6.48
	Academic center	3	2.78
	Academic hospital (acute care)	3	2.78
	Academic hospital (inpatient rehabilitation)	3	2.78
	Academic hospital (NICU)	2	1.85
	Home health agency	2	1.85
	Childcare center	2	1.85
	Community hospital (acute care)	1	0.93
	Community hospital (NICU)	1	0.93
	Other	1	0.93
Percentage caseload 0–3 years (*n* = 67)	1–25	9	13.43
	26–50	8	11.94
	51–75	11	16.42
	76–100	39	58.21
How many children < 3 years with CP seen in typical month (*n* = 70)	0	8	11.43
	1–5	43	61.43
	6–10	8	11.43
	11–15	6	8.57
	16–20	1	1.43
	21–25	2	2.86
	26–30	1	1.43
	31–35	1	1.43
Membership in professional societies or groups (*n* = 76) (61 individual respondents)	No memberships	33	43.42
	APTA	13	17.11
	AOTA	12	15.79
	AACPDM	6	7.89
	Child Neurology Society	3	3.95
	AAPM&R	1	1.32
	POSNA	1	1.32
	Other	7	9.21

* Percentages calculated according to number of responses (not number of individual respondents). ^#^ Indicates professions that may diagnose cerebral palsy within their scope of practice. AACPDM: American Academy of Cerebral Palsy and Developmental Medicine; AAPM&R: American Association of Physical Medicine and Rehabilitation; AOTA: American Occupational Therapy Association; APTA: American Physical Therapy Association; NICU: neonatal intensive care unit; POSNA: Pediatric Orthopaedic Society of North America.

**Table 2:** Reasons for non-use of GMA or HINE (respondents could select multiple answers).

GMA (*n* = 48)	HINE (*n* = 41)
I don’t know how to administer and/or score, *n* = 32	I don’t know how to administer and/or score, *n* = 29
My workplace does not support it, *n* = 12	My workplace does not support it, *n* = 10
I rely on clinical signs and symptoms, *n* = 10	I rely on clinical signs and symptoms, *n* = 12
It is out of my scope of practice, *n* = 3	It is out of my scope of practice, *n* = 3
I use another assessment tool, *n* = 4	I use another assessment tool, *n* = 2
I have been trained but I don’t feel comfortable using, *n* = 1	I have been trained but I don’t feel comfortable using, *n* = 2
Early detection of CP not part of my practice pattern, *n* = 1	Early detection of CP not part of my practice pattern, *n* = 2
Unsure about its effectiveness, *n* = 1	Unsure about its effectiveness, *n* = 0
Too expensive, *n* = 2	Too expensive, *n* = 0
Too time consuming, *n* = 1	Too time consuming, *n* = 1
Other reasons, *n* = 5	Other reasons, *n* = 5
Other team members perform in clinic, *n* = 1	Other team members perform in clinic, *n* = 1
I am not familiar with the tool, *n* = 3	I am not familiar with the tool, *n* = 1
I don’t do this, *n* = 1	Time needed for other required tests, *n* = 1
	I do a standard neurological exam, *n* = 2

CP: cerebral palsy; GMA: General Movement Assessment; HINE: Hammersmith Infant Neurological Examination.

## Data Availability

The data set for this study may be found at: https://osf.io/hr5qx/ (accessed 14 March 2024).

## Supplementary Materials

The following supporting information can be downloaded at: https://www.mdpi.com/article/10.3390/children11050515/s1, Supplementary File S1: Survey questions; Supplementary Table S1: Age of diagnosis free-text examples; Supplementary Table S2: Barriers and Enablers free-text examples.
