# Supplementary material for: Identifying Opportunities for Early Detection of Cerebral Palsy
Source: Children (Basel). 2024 Apr 25;11(5):515. doi: 10.3390/children11050515 (PMC11119564; doi:10.3390/children11050515)
Supplement: Supplementary file 1 [file children-11-00515-s001.zip › Supplementary File S1.pdf]

Clinician survey: Early detection and intervention of infants with or at risk of Cerebral Palsy across Delaware and Maryland

This is a voluntary research survey targeted towards all health professionals across Delaware and Maryland working with young children (3 years and younger) who have or are at risk of CP including genetic diagnoses that result in lifelong non-degenerative sensorimotor impairments (ex. Angelman syndrome, Chri du Chat, etc.), metabolic disorders, anatomic anomalies (holoprosencephaly, etc.) and other causes of CP (IVH, PVL, HIE, etc.).

To be eligible for this study, health professionals must currently (or within the previous year) provide services (either education or healthcare) in the states of MD or DE to children from birth to age 3 who present with risk factors/criteria for Cerebral Palsy (CP). This includes health professionals from across both states working with children with CP, specifically targeting Neonatologists, Pediatricians, Neurologists, Nurses, Orthopedic Surgeons, and Allied Health Professionals.

When you see the word diagnosis it could mean that you are diagnosing or referring to someone that would diagnose cerebral palsy.

Voluntary Consent: You are being asked to complete a survey as part of a research study. It is up to you whether you choose to participate or not.

Purpose: The aim of this study is to evaluate current practices regarding diagnosis and referral practices for early intervention for children (birth - 3 years) at risk or diagnosed with cerebral palsy (CP).

Duration: 10 - 15 minutes.

Risks: To minimize the risk of loss of confidentiality, no PHI will be collected and the data obtained will be anonymous.

Benefits: There are no direct benefits to participating in this study. However, your participation will help us determine specific education and resource needs across different regions of Delaware and Maryland, which could then be used to facilitate consistent practices for early diagnosis of CP across the state of Maryland and Delaware.

Contact info: If you have questions prior to consenting, you can contact the Brittany Hornby at hornby@kennedykrieger.org or the PI, Wade Shrader at wade.shrader@nemours.org.

If you have questions about your rights as a research participant, what to do if you are injured, if you would like to offer input or obtain information, or if you cannot reach the investigator or want to talk to someone else who is not involved with this research, you may contact the persons listed below.

Chairperson, Nemours IRB 2 at 904-697-3415
Director, Nemours Office of Human Subjects Protection at 302-298-7613
Email address: NOHSP@nemours.org

We are hoping to reach as many practictioners in the states of DE and MD as possible, so we would appreciate if you could share this survey with your colleagues and staff.

Research Consent

Please review the information above. Do you consent to taking part in this anonymous voluntary research survey?	☐ I consent to take part in this study.

A little bit about you (1/3)

What is your current profession? Select all that apply.

- ☐ Developmental Pediatrician
- ☐ Early childhood nurse
- ☐ Early Intervention Administrator
- ☐ General Pediatrician
- ☐ General Practitioner
- ☐ Neonatologist
- ☐ Nurse practitioner/Physician Assistant
- ☐ Nutritionist/Dietitian
- ☐ Occupational Therapist
- ☐ Occupational Therapist Assistant
- ☐ Orthopedic Surgeon
- ☐ Orthotist (Bracing)
- ☐ Pediatric Neurologist
- ☐ Pediatric Rehabilitation Physician (Physiatrist)
- ☐ Physical Therapist (Physical Therapist)
- ☐ Physical Therapist Assistant (Physical Therapist Assistant)
- ☐ Psychologist
- ☐ Researcher/Scientist
- ☐ Social Worker
- ☐ Special Educator (Teacher)
- ☐ Speech Language Pathologist
- ☐ Developmentalist/Developmental Therapist
- ☐ Trainee Doctor
- ☐ Other

If other profession, please specify

____

How many years of experience do you have working in the field of pediatrics?

- ◯ None
- ◯ 1-5 years
- ◯ 6-14 years
- ◯ 15 or more years

Describe the setting that you work in. Select all that apply.

- ☐ Community Hospital (acute care)
- ☐ Community Hospital (NICU)
- ☐ Community Hospital (inpatient rehabilitation)
- ☐ Academic Hospital (acute care)
- ☐ Academic Hospital (NICU)
- ☐ Academic Hospital (inpatient rehabilitation)
- ☐ Outpatient based community hospital center
- ☐ Outpatient based academic hospital center
- ☐ Private practice
- ☐ Early Intervention/Infants and Toddlers under Part C
- ☐ NICU Follow up Program
- ☐ Home health agency
- ☐ Schools (education services)
- ☐ Childcare Center
- ☐ Academic Center (example: University/college)
- ☐ Other

If other setting, please specify.

____

Community hospital NICU: What level NICU do you work in?

- ◯ Level I
- ◯ Level II
- ◯ Level III

Academic hospital NICU: What level NICU do you work in?	◯ Level I<br>◯ Level II<br>◯ Level III
At what age do typically suspect cerebral palsy, without sharing this with the family or formally give a diagnosis, in children in your care?	◯ 0-6 months<br>◯ 7-12 months<br>◯ 13-24 months<br>◯ 25-36 months<br>◯ > 36 months
Explanation (optional): At what age do children in your care typically receive the diagnosis of cerebral palsy from you or another provider?	◯ 0-6 months<br>◯ 7-12 months<br>◯ 13-24 months<br>◯ 25-36 months<br>◯ > 36 months<br>◯ Other
If other age, please specify.	__________________________________
Do you have any thoughts or comments on the current age of diagnosis or detection of CP of children in your service? Explanation (optional)	__________________________________
Are you a member of any professional societies/groups? Select all that apply.	☐ No<br>☐ American Academy of Cerebral Palsy & Developmental Medicine (AACPDM)<br>☐ American Physical Therapy Association (APTA)<br>☐ American Occupational Therapy Association (AOTA)<br>☐ American Speech and Hearing Association (ASHA)<br>☐ American Academy of Physical Medicine and Rehabilitation (APMR)<br>☐ Association of Academic Physiatrists (AAP)<br>☐ Pediatric Orthopedic Society of North America (POSNA)<br>☐ Child Neurology Society (CNS)<br>☐ Other
If you are a member of another professional society/group, please specify.	__________________________________

In which county (counties) is (are) your current place of employment? Select all that apply.	☐ Allegany<br>☐ Anne Arundel<br>☐ Baltimore City<br>☐ Baltimore County<br>☐ Calvert<br>☐ Caroline<br>☐ Cecil<br>☐ Charles<br>☐ Dorchester<br>☐ Frederick<br>☐ Garrett<br>☐ Harford<br>☐ Howard<br>☐ Kent<br>☐ Montgomery<br>☐ New Castle<br>☐ Prince George's<br>☐ Queen Anne's<br>☐ St. Mary's<br>☐ Somerset<br>☐ Sussex<br>☐ Talbot<br>☐ Washington<br>☐ Wicomico<br>☐ Worcester<br>☐ Other
If you are employed in another county not listed, please specify.	________________________________
Percentage of caseload treating children 0-3 years.	0 50 100<br>*(Place a mark on the scale above)*
How many children do you typically see with CP under age 3 in a usual month?	◯ 0 ◯ 1-5 ◯ 6-10<br>◯ 11-15 ◯ 16-20 ◯ 21-25<br>◯ 26-30 ◯ 31-35 ◯ 36-40<br>◯ 41-45 ◯ 46-50 ◯ >50

Diagnosis and detection of CP (2/3)

If you suspect that a patient may have or be at risk of cerebral palsy, what is your usual referral pathway? Select all that apply.

- ☐ Pediatric Neurology
- ☐ General Pediatrics
- ☐ Developmental Pediatrics
- ☐ Pediatric Orthopedics
- ☐ Physical Medicine and Rehabilitation (Physiatrist)
- ☐ Therapies PT, OT, SLP
- ☐ Other
- ☐ I usually do not refer on because

____

If you selected other referral pathway, please specify.

____

If you selected "I usually do not refer on because _____" please specify.

____

The typical wait for this referral appointment is

- ◯ 1-6 days
- ◯ 1-2 weeks
- ◯ 1-2 months
- ◯ 3+ months

Does your workplace provide any standard guidelines or procedures for referring a child for diagnosis for early detection of a risk of cerebral palsy?

- ◯ Yes
- ◯ Yes, but they could be improved
- ◯ No, but I have my own
- ◯ No
- ◯ Unsure
- ◯ Other

If you selected other guidelines for referral, please specify.

____

How often do you personally use these assessment tools for children with or at risk for cerebral palsy?

	Never	Sometimes	Almost always	Not applicable to the ages I work with
Alberta Infant Motor Scale (Birth-18 months)	☐	☐	☐	☐
Bayley Scales of Infant and Toddler Development (1-42 months)	☐	☐	☐	☐
Cranial ultrasound (birth-6 months)	☐	☐	☐	☐
Developmental Assessment of Young Children (Birth-5 years)	☐	☐	☐	☐
Dubowitz Neurological assessment (pre-term to 2 months)	☐	☐	☐	☐
Hammersmith Infant Neurological Examination (2-24 months)	☐	☐	☐	☐
Magnetic Resonance Imaging of the brain (all ages)	☐	☐	☐	☐
Motor Assessment of Infants (2 months-2 years)	☐	☐	☐	☐
Neuro Sensory Motor Development Assessment (1 month-6 years)	☐	☐	☐	☐
Prechtl's Qualitative Assessment of General Movements (birth-20 weeks)	☐	☐	☐	☐
Peabody Developmental Motor Scales-2 (Birth- 5 years)	☐	☐	☐	☐
Test of Infant Motor Performance (34 weeks gestational age-4 months post term corrected age)	☐	☐	☐	☐
Genetic testing (all ages)	☐	☐	☐	☐
Other (please specify)	☐	☐	☐	☐

Other assessment tool __________________________________

What are the reasons why you do not use the Hammersmith Infant Neurological Examination? Select all that apply.	☐ I don't how administer and/or score the HINE<br>☐ I have been trained but I don't feel comfortable using the HINE<br>☐ Early detection of CP is not part of my practice pattern<br>☐ Unsure about its effectiveness<br>☐ It is out of my scope of practice<br>☐ Too expensive<br>☐ Too time consuming<br>☐ Families don't like it<br>☐ Lack of staff<br>☐ My workplace does not support it<br>☐ Not wanting to be the provider responsible for conveying this information to family<br>☐ I rely on clinical signs and symptoms<br>☐ I use another assessment tool<br>☐ Other
Other reason for not using HINE	__________________________________
What are the reasons why you do not use (or refer the child to) Magnetic Resonance Imaging of the brain for children under 1 year of age? Select all that apply.	☐ Unsure about its effectiveness<br>☐ Lack of resources<br>☐ Not appropriate for the age group I see<br>☐ Too expensive<br>☐ Too time consuming<br>☐ Families don't like it<br>☐ Lack of staff<br>☐ My workplace does not support it<br>☐ Not familiar to me<br>☐ Other
Other reason for not using MRI	__________________________________
What are the reasons why you do not use Prechtl's Qualitative Assessment of General Movements? Select all that apply.	☐ I don't how administer and/or score the GM<br>☐ I have been trained but I don't feel comfortable using the GM<br>☐ Early detection of CP is not part of my practice pattern<br>☐ Unsure about its effectiveness<br>☐ It is out of my scope of practice<br>☐ Too expensive<br>☐ Too time consuming<br>☐ Families don't like it<br>☐ Lack of staff<br>☐ Not wanting to be the provider responsible for conveying this information to family<br>☐ My workplace does not support it<br>☐ I rely on clinical signs and symptoms<br>☐ I use another assessment tool<br>☐ Other
Other reason for not using Prechtl's Qualitative Assessment of General Movements	__________________________________

When you have given the HINE, what do you next? (select all that apply)	☐ I didn't know there was something else I needed to do<br>☐ I know how to get the total score<br>☐ I know how to look up the score and see if a kid is at risk for CP or not<br>☐ I know how to predict GMFCS at age 2<br>☐ Other/explain
Other/explain after giving HINE	__________________________________
In regards to the Hammersmith Infant Neurological Examination, how to score, and the optimality scoring of this tool (i.e. the cut off scores for the risk of CP and the risk of non-ambulant CP), please select one:	○ Yes, I am familiar with the tool, how to score and the optimality scores<br>○ Yes, I am familiar with the tool and how to score, but not the optimality scores<br>○ I am familiar with the tool but not the scoring<br>○ No, I am not familiar
When I use GMA I know how to: (select all that apply)	○ I know how to assess general movements before 2 months adjusted age (writhing period)<br>○ I know how to identify cramped synchronous movements/poor repertoire.<br>○ I know how to administer general movement optimality score for birth to 2 months adjusted age (writhing period)<br>○ I know how to identify abnormal/absent fidgety movement 2-5 months adjusted age<br>○ I know how to administer the Motor Optimality Score for 2-5 months adjusted age

What clinical signs and symptoms would prompt a referral for diagnostic assessment of cerebral palsy? Select all that apply and how often they prompt a referral.

	Almost never	Rarely	Not so frequently	Somewhat frequently	Very frequently	Most frequently
Abnormal reflexes	◯	◯	◯	◯	◯	◯
Excessive head lag	◯	◯	◯	◯	◯	◯
Repetitive open and closing mouth	◯	◯	◯	◯	◯	◯
Atypical finger posture	◯	◯	◯	◯	◯	◯
Monotonous kicking	◯	◯	◯	◯	◯	◯
Cramped synchronized movement	◯	◯	◯	◯	◯	◯
Circular arm movement with finger spreading	◯	◯	◯	◯	◯	◯
Repetitive kicking without variation	◯	◯	◯	◯	◯	◯
Persistent fisting in babies less older than 4 months of age	◯	◯	◯	◯	◯	◯
Persistent inclination of head/trunk	◯	◯	◯	◯	◯	◯
Poor head control in babies older than 4 months	◯	◯	◯	◯	◯	◯
Delayed sitting without support after 9 months	◯	◯	◯	◯	◯	◯
Stiffness/tightness in legs between 6-12 months	◯	◯	◯	◯	◯	◯
Early handedness before 12 months	◯	◯	◯	◯	◯	◯
Any asymmetry in posture or movement	◯	◯	◯	◯	◯	◯
Whole body contracting into bent posture that causes baby to get upset or cry	◯	◯	◯	◯	◯	◯
Other	◯	◯	◯	◯	◯	◯

What other signs and symptoms prompt a referral for CP? __________________________________

Were you aware that cerebral palsy can frequently be diagnosed by 12 months of age?

◯ Yes
◯ No

Change in practice (3/3)

In your current workplace, what facilitators help you to implement new assessment tools/change of practice?

In your current workplace, what barriers prevent you from implementing new assessments tools / change of practice?

If you have anything further to add about the topics covered in this survey, please respond below.
