# Supplementary material for: Identifying Opportunities for Early Detection of Cerebral Palsy
Source: Children (Basel). 2024 Apr 25;11(5):515. doi: 10.3390/children11050515 (PMC11119564; doi:10.3390/children11050515)
Supplement: Supplementary file 1 [file children-11-00515-s001.zip › Supplementary Table S1.pdf]

Supplementary Table S1: Respondent experiences regarding age of diagnosis in Maryland and Delaware

**Delayed diagnosis common (n = 15)**	
Too late, Doctor reluctance, Wait and see approach	We wait too long! ID50
	It is often diagnosed much later than it should be ID45
	diagnosis could be earlier, MDs often hesitant to label too quickly, it seems ID56
	The doctors tend to be reluctant to diagnose CP early and usually will say "let's wait and see" ID7
**Some diagnosed early (n = 4)**	
	Some babies are diagnosed w/CP younger than 7 months ID45
	Early detection recommended as would also select 0-6 months above as well ID20
**Age of diagnosis influenced by severity (n = 3)**	
Timing of diagnosis	depends upon the severity. there are kids who suspect at 0-6m given their presentation and history and may receive a diagnosis that early or 7-12 mo, but more often it is not until kids are not walking by age expected milestone ID 31
	They either get a CP dx at birth or it will not be until 18+ months, usually between 18 and 30 months ID 29
	milder cases of diplegia or hemipares, often is delayed to 25 - 36+months ID 92
**System factors: Referral and health pathways (n = 20)**	
Diagnostic test limitations	Usually hear the doctors do not want to do testing (that may or may not involve sedation) until 2 years of age ID32
Referral delays	delays in or barriers to getting genetic testing or getting sedated MRI may contribute to perceived delayed diagnosis ID53
Specialty clinics versus community	I think our specialty clinics do a good job of diagnosing CP early. I think there are many patients in the community that come to us later without having been given a Dx ID61
Challenges	It is more challenging to get a diagnosis of CP now compared to 20 years ago ID80
**Social factors: Family perceptions and attitudes (n = 5)**	
Family impact of delay	The longer diagnosis is deferred, the more difficult for parents and family ID49
Family acceptance/readiness	diagnosis sometimes takes a while due to other health issues, difficulty accepting that CP may be present by the family, and wanting to rule out other possible explanations first. ID73
	working on improving - balancing goal of early diagnosis with individual family need/readiness (without limiting therapies/support) ID34
Parent, provider driven	Feels children are being treated to address deficits but not getting a formal diagnosis until later unless parents or providers push for a diagnosis ID6
**Health provider knowledge and perceptions (n = 12)**	
Lack of multidisciplinary working<br>Resistance to change<br>Lack of knowledge	I wish MDs would identify earlier and trust providers ... stop recommending more of the same therapy and identify resources ID41
	Yes it is too late in coming when we see signs early enough to discuss much sooner than the pediatrician is willing to Id5
	I find that I'm frequently the first professional to discover that they probably have CP. ID95
Early 'at risk' diagnosis	I like to diagnose or at least educate families on the possible diagnosis of CP so they can get the correct services in place ID37
Health provider perceptions/bias	"CP" is such a broad swatch. For the mildest i tend to use another label in medical communications because of the potential for mis-interpretation of its impact on functioning ID48
