# Supplementary material for: Identifying Opportunities for Early Detection of Cerebral Palsy
Source: Children (Basel). 2024 Apr 25;11(5):515. doi: 10.3390/children11050515 (PMC11119564; doi:10.3390/children11050515)
Supplement: Supplementary file 1 [file children-11-00515-s001.zip › Supplementary Table S2_March_15-2024.pdf]

Supplementary Table S2: Enablers and barriers to use of new tools/assessment processes in Maryland and Delaware

System Factors	
**Enablers (n = 13)**	**Barriers (n = 44)**
Funding	
leadership support to purchase assessment, training in new assessment for qualified examiners, training for entire program in referral pathway for suspected CP ID72	Insurance Reimbursement for eval and ergo profits for training/purchase. ID100
Tool availability and use	
Test availability or ability to request/order new tests as needed, admin support, info/support from pediatric section/conferences ID50	recently, cost and online features only of newly revised assessment tools ID20
It is up to individual practitioners when related to CP ID69	Learning how to administer the assessment and having scoring information. Cost of the testing materials ID49
Time, workload, staffing	
using them, demonstrating value. having time to do them. ID53	Time/effort to update processes ID2
	Financial, time constraints. Lesser so resistance/reluctance of staff to change/implement. ID34
	having such a large staff, time to practice and be trained ID82
	Heavy workload, focus on broad early intervention rather than specific areas of need ID35
	At the initial we are conducting testing in 7 areas and a Routines Based Interview for children under 12 months so time is the major factor in adding additional tests. ID80
Organizational structure/processes	
none, I am free to use what I find fit. ID92	Assessment tools are approved by the administration in the Department of Health and Human Services and they do the reviews of what tools we use. Then our software has to be changed to reflect this. So changes in tools are made very infrequently only by administration. We can make recommendations and they will be reviewed. We could be asked to present the tool to see if it is more accurate and efficacious than current tools.ID73
	State of MD has preferred tools for assessment. Those are the ones typically paid for, bought and taught to staff for use. ID81
	they have to be approved by the county - typically the head of the department (OT or PT) has to be a part of presenting it to the county board and being sure it meets criteria ID31
	Changes in policy regarding access to and use of standardized assessment tools is solely controlled at an administrative level with little to no input from clinicians.ID26
	Clinical support staff are not available, primitive computer access with poor consistency, lack of

	office space/storage space, and lack of administrative support of clinic ID57
**Social Factors**	
**Enablers (n = 25)**	**Barriers (n = 21)**
Administration/Leadership	
NICU follow up clinic providers or director of PT ID 20	Lack of administrator support, primary service provider model, system requirements ID4
The supervisor team, including the director ID29	Administration...our IT program's lead agency is the public school system and sometimes do not value our medical background as a basis to make any changes. ID5
leadership support to purchase assessment, training in new assessment for qualified examiners, training for entire program in referral pathway for suspected CP ID72	leadership by in ID22
Peer support/Multi-disciplinary working/Clinical champions	
My PT colleague ID21	Practicing in a small rural county by myself ID21
Coworkers advocating ID22	*Resistance to change*
Would be a meeting of all the PT's (for example) to bring to administration our concern or strong belief in all getting HINES trained (as an example)! ID5	Habit, cost of assessment tools, time of some tests ID94
As a Nemours physical therapist, We have a CP work group with a team leader who helps to implement new assessment tools or change of practice ID46	Too busy to slow down and learn a new tool. ID45<br>a feeling of "this is always how we have done it" ID87
**Health provider knowledge and perceptions**	
**Enablers (n = 22)**	**Barriers n = 19**
Access to education	
Limited - currently working to get HINE training for physicians, NP/PAs, therapists - would also love to get GMA training for team members.ID34	Training/knowledge of tool ID28<br>Lack of access, training, or mentorship on these tools/topics ID36
Knowledge sharing/confidence/practice opportunities	
State Department Research evidence ID 6	
inform other early interventionists (besides motor therapists) about common concerns that could align with CP indicators ID82	We have access to many assessment tools and can choose which one we want to use. I do not have extensive training and do not feel confident enough to use all of them, therefor, I end up using the same ones over and over again. ID97
Training and practice prior to implementing Id56	equipment, the time needed when learning a new assessment - prep time to review new assessment, education on new assessment tool, increased time when performing a new assessment with patient, etc ID46
Guidelines and Pathways	
Guidelines from MSDE for Part C ID25	Lack of time, training, and opportunity to practice ID56<br>time to learn new tools, comfort level ease of using "what's familiar" ID61
**Clinical considerations and Internal drive**	
**Enablers (6)**	**Barriers (n =4)**
WE can use any assessment that we feel is clinically appropriate ID49<br>I would do the assessments in my own. ID77	length of assessment and practicality ID93
